# Evidence‐Based Nursing Competence, Attitudes, and Associated Factors Among Nurse Leaders in Finland: A Cross‐Sectional Study

**DOI:** 10.1155/jonm/4526663

**Published:** 2026-05-26

**Authors:** Saija Ylimäki, Jemina Qvick, Anna-Maria Tuomikoski, Heidi Parisod, Hannele Siltanen, Outi Kanste, Maria Kääriäinen

**Affiliations:** ^1^ Research Unit of Health Sciences and Technology (HST), Faculty of Medicine, University of Oulu, Oulu, Finland, oulu.fi; ^2^ Medical Research Center Oulu, University Hospital and University of Oulu, Oulu, Finland, oulu.fi; ^3^ The Finnish Centre for Evidence-Based Health Care, A Joanna Briggs Institute Centre of Excellence, Helsinki, Finland; ^4^ Wellbeing Services County of North Ostrobothnia, Oulu University Hospital, Oulu, Finland, ppshp.fi; ^5^ Nursing Research Foundation, Helsinki, Finland

**Keywords:** attitudes, competence, evidence-based nursing, leaders, nursing, supporting structures

## Abstract

**Aim:**

To describe evidence‐based nursing (EBN) competence, attitudes, and associated factors among a sample of Finnish nurse leaders.

**Design:**

This study was carried out as a descriptive cross‐sectional study.

**Methods:**

Data were collected via an electronic questionnaire in 2021 from 215 nurse leaders registered in nursing trade unions, working as front line, middle, or senior leaders in Finnish social and healthcare organizations. A self‐assessment instrument (ActEBN‐managers) was used to measure EBN competence, attitudes, and supporting structures. Data were analyzed using descriptive statistics, *t*‐test, analysis of variance, and linear regression. The reliability of the ActEBN‐managers instrument was 0.893.

**Results:**

In this sample of Finnish nurse leaders, EBN competence and attitudes were overall good. EBN competence and attitudes were associated with several background factors, as well as with factors related to organizational support structures and culture, in the independent group analyses. An independent statistical association was identified between EBN competence and higher education (*t* = 2.074, *p* = 0.039). Furthermore, an independent statistical association was identified between EBN attitudes and the service provider background (*t* = −2.503, *p* = 0.013), with more positive attitudes observed among nurse leaders working in the public sector. EBN attitudes were also associated with the availability of a best practices bank (*t* = −2.591, *p* = 0.010).

**Conclusions:**

The findings suggest that social and healthcare organizations may benefit from focusing on strengthening nurse leaders’ EBN competence and attitudes by supporting their educational development, introducing EBN support structures more widely, and promoting an organizational culture that facilitates EBN. Nurse leaders play an important role in enabling and supporting the implementation of EBN.

**Summary**
• Highlights
◦ The EBN competence of nurse leaders in this sample warrants closer examination, as notable variation was identified in their reported EBN competence and attitudes.
◦ Higher educational attainment was independently associated with higher EBN competence within this sample of nurse leaders.
◦ EBN attitudes were independently associated with the service provider background in this study sample, with more positive attitudes observed among nurse leaders working in the public sector.
◦ EBN attitudes were also associated with the availability of a best practices bank as part of the organization’s EBN support structures within this sample.
• Implications for the Profession
◦ EBN competence development for nurse leaders should be targeted primarily at front‐line leaders with lower levels of education and working in private social and healthcare.
• Impact
◦ The study suggests that EBN competence among Finnish nurse leaders varies and may require further development. Higher education and organizational support can enhance EBN competence and attitudes among nurse leaders at different levels and settings. Organizations should strengthen nurse leaders’ EBN competence through graduate‐level education, implementation of EBN support structures, and an organizational culture that prioritizes EBN.
• Reporting Method
◦ STROBE guidelines were followed in reporting.

## 1. Introduction

Healthcare should be based on scientifically proven evidence. In addition, the treatment and practices must be safe, of high quality, and properly implemented [1, 2]. Effective, relevant, high‐quality, and safe care for patients; improved patient outcomes; and lower costs in healthcare [3, 4] are linked to evidence‐based healthcare (EBHC). EBHC constitutes a comprehensive process that includes the identification of knowledge needs, the generation and synthesis of evidence, the translation and transfer of evidence, and its implementation into nursing practice. The evidence is systematically compiled, up to date, and critically evaluated data. The concept of evidence‐based practice (EBP) refers to the use of the best available evidence in decision‐making related to patient care, while also considering the healthcare context, customer’s preferences, and the judgment of a healthcare professional. Its purpose is to allow uniform and safe treatment regardless of the healthcare provider [2]. Although the significance of EBP is well known [5], low evidence usage is a challenge both nationally and internationally [5, 6]. EBP that incorporates the stages of the EBHC model and is applied in nursing is referred to as evidence‐based nursing (EBN).

Nurse leaders in the front‐line, middle, or senior leader and managerial positions of healthcare organizations are responsible for the management of their organizations, such as the competence of the nursing staff, finances, ensuring quality care practices and patient safety. In view of these responsibilities, the nurse leader will have the greatest impact on EBN [7]. They play an important role in putting the evidence into practice, developing organizational supporting structures, and creating an organizational culture that supports the EBN [7, 8]. However, their own competence of EBN and the low use of evidence have been identified as one of the challenges to implement evidence [8–10], which is reflected in the competence of the entire organization’s EBN and the use of the evidence of the nursing staff. This requires the identification of nurse leaders’ EBN competence and attitudes [7, 8] and associated factors [7].

Evidence implementation requires analyzing the context, promoting change, and evaluating processes and results [2]. Introducing new practices or changing existing practices within the organization requires changes in the behavior of the relevant actors [11]. The key to successful implementation is to identify and understand the importance of leadership, organizational support, and professional culture in this process [12]. The aim is to better understand and explain why and how the implementation will succeed or fail. The more detailed the behavior is, the easier it is to identify the promoters and inhibitors of change [11].

In Finland, leadership and management of nursing are based on experience in the field, nursing and management expertise, and knowledge of the social and healthcare environment and the service system. At a higher level, nurse leaders are recommended to require an undergraduate degree in health, an appropriate higher university degree, and management and administration experience and training. To provide a supportive environment and organizational culture and to ensure evidence implementation, the nurse leaders must have sufficient competence in EBN [8].

For the purposes of this study, EBN competence refers to nurse leaders’ characteristics, knowledge, and responsibilities related to EBN and its promotion. Nurse leaders must be proactive, expert, supportive, and persistent in relation to EBN [13]. They are required to have an extensive knowledge of the EBHC model and EBN and its management to take responsibility for improving treatment and clinical and economical outcomes [6]. It is important to understand their role in creating and supporting an optimal environment [14]. They can promote EBN by their own example and by seeking reform structures, processes, and work culture. They must be able to support nursing staff in EBN and ensure the necessary human and other resources [15] such as time for the EBN [15, 16]. In addition, the nurse leaders should be able to transfer evidence by providing nursing staff with access to EBN education and by developing the roles of EBN specialists and mentors [15]. As a whole, they need to be able to identify development and information needs, develop and use supporting structures of EBN, and enable the change of practices, evidence implementation, evaluation, and consolidation [13, 15].

However, the nurse leaders have had a lack of knowledge and experience of EBN [8]. Still, there are wide differences in competence in EBN among nurse leaders due to different educational backgrounds [6]. Their own training, professional title, and postgraduate studies are linked to competence in EBN [6, 8, 14]. The nurse leaders’ competence in EBN can be supported through various interventions and strategies, e.g., education [7, 8]. The developing competence in EBN can strengthen EBN leadership and promote the implementation on EBN within organizations, ultimately improving patient outcomes [7].

In addition to competence in EBN, nurse leaders’ attitudes toward EBN are crucial. Previous studies have found that nurse leaders understand the importance of EBN [8, 10] and their attitudes toward EBN may be positive [8], but they tend to be passive about EBN [6, 10]. Although the main priorities of nurse leaders are safety and quality of care, EBN is not considered a priority. This demonstrates the importance of the introduction of evidence by nurse leaders in order to achieve safety and quality [10].

Organizational support structures refer to the structures that support the implementation of EBN. These may include different organizational infrastructure, education and mentoring, and multiprofessional cooperation and expertise [17]. In this study, the supporting structures of EBN refer to the models, care bundles, EBPs, and auditing to support EBN in nursing. Action Model of Expertise (FinAME) is used to identify the roles and activities of different clinical nursing experts, nurse leaders, educators, and researchers in promoting EBN. It helps nurse leaders to support different nursing experts in EBN even if the titles, roles, and tasks of different nursing experts in healthcare are inconsistent. The Model for Developing Evidence‐Based Practices (OMEBP) describes responsibilities in the development and implementation of consistent EBPs at different healthcare system levels. It helps to decrease unjustified variations in clinical care and enable the best possible care regardless of the healthcare provider [18].

In contrast, an evidence‐based organizational culture consists of a shared will and commitment of health professionals and managers to promote, support, and implement EBPs [8], and has associations for the development of EBN support structures. Organizational culture can have a significant impact on the success of EBN, depending on its openness and readiness to change [19]. Without a culture supporting EBN, EBN is unlikely to be sustainable [5]. The role of nurse leaders is to create an organizational culture that supports EBN [15]. They should develop a shared understanding of EBN within the organization and highlight the importance of EBN and its impacts [16]. However, one of the challenges for EBN has been the lack of an organizational culture supporting EBN [5].

Some previous studies examined leader EBN competence, attitudes, and barriers, yet the link between specific organizational support structures and leaders’ competence and attitudes has not been quantified in a national sample.

## 2. The Study

The aim of the study was to describe EBN competence, attitudes, and associated factors among nurse leaders in Finland. The objective was to generate information to support the development of EBN competences of nurse managers and the creation of organizational strategies to promote them.

The research questions were the following:

1. What are the EBN competence and attitudes among nurse leaders?
2. How are the background characteristics of nurse leaders associated with EBN competence and attitudes?
3. How are organizational factors (such as support structures and organizational culture) associated with EBN competence and attitudes among nurse leaders?

## 3. Methods

### 3.1. Study Design

The study was designed as a cross‐sectional study and carried out in Finland as part of a wider national study assessing the state of EBN. The estimated target population consisted of approximately 5300. A total of 215 responses were received, and the response rate was approximately 4%. Because only a small proportion of the target population participated, the representativeness of the sample is limited, and the findings should be interpreted cautiously. Given the low response rate, the precision of the estimate was assessed using a 95% confidence interval calculated with the OpenEpi calculator. The mean score was 4.01 (SD = 0.48, *n* = 215), with a 95% confidence interval of 3.947–4.072, corresponding to a precision of ±0.063 units.

### 3.2. Study Participants

The target population of this comprehensive study was all the nurse leaders who were registered members of the nursing associations and unions in the field of nursing in Finland. The inclusion criteria were as follows: working as front‐line, middle, or senior leader in public or private social and healthcare organization and any nursing settings (e.g., hospital, healthcare center, homecare).

### 3.3. Instruments

The study used the ActEBN‐managers instrument, which measures attitudes, competence, and organizational culture in EBN. In addition, the study included items mapping EBN support structures and background characteristics questions. The ActEBN‐mangers instrument has not yet undergone full psychometric validation. At the current stage, validation activities have included content and face validity assessment by national expert panel, pilot testing, and evaluation of internal consistency reliability. Construct validity testing, such as confirmatory factor analysis (CFA), has not been performed, and therefore the results should be interpreted with this limitation in mind.

The ActEBN‐managers instrument was developed by the Finnish Nursing Research Foundation (NRF) and the University of Turku [20]. Indicators on the subject were mapped through a literature review in 2019. The theoretical frameworks for developing the instrument were JBI’s Model of Evidence‐based healthcare [2] and OMEBP [21]. In addition, the items were included from the EBP process assessment scale [22] to measure attitudes and competence in EBN. The developers of the original instrument obtained written permission to translate and use the items [20].

The content and face validity of the developed instrument was assessed nationally by EBN experts from different organizations (*n* = 15) in 2020. The scope of the different items of the instrument and the clarity and relevance of items were assessed. The instrument was modified based on expert judgments. The instrument was piloted in spring 2021. Nurses working in clinical practice (ActEBN‐nurses) instrument have been validated, and the instrument for nurse leaders (ActEBN‐managers) was developed to respond to their different roles [20].

The ActEBN‐managers instrument contains 12 items on competence, 16 items on attitudes, and 7 items on organization culture (Table 1). Items on EBN competence included questions on identifying one’s own role, critically evaluating current practice and research evidence, using evidence to develop practice, and evaluating nursing outcomes. Items on EBN attitudes included questions on how respondents assessed the impact and relevance of evidence and EBP in nursing. The items on competence, attitudes, and organization culture were measured with the 5‐point Likert scale (1 = completely disagree, 5 = fully agree).

**TABLE 1:** Mean, standard deviation, and Cronbach’s alpha of the sum variables.

Sum variable	Mean (SD)	95% CI	Score min–max	Cronbach’s alpha	*n*
Competence^∗^ (12 items)	4.01 (0.476)	3.95–4.08	2.25–5.00	0.898	215
Attitudes^∗^ (16 items)	4.17 (0.413)	4.12–4.23	2.94–5.00	0.841	215

*Note:* CI, confidence interval for mean.

Abbreviation: SD, standard deviation.

^∗^Subscale‐level results are not applicable because the ActEBN‐managers instrument comprises single‐dimension competence and attitude scales without predefined sub‐dimensions.

The internal consistency of the ActEBN‐managers instrument based on Cronbach alpha (α) was 0.893 (Swedish *α* = 0.927, Finnish *α* = 0.890), varying from 0.841 to 0.898 (Swedish 0.900–0.939, Finnish 0.833–0.890*α*) by sub‐dimensions (Table 1). Comparisons between language versions should be interpreted with caution, despite the instrument’s excellent reliability, as formal measurement invariance testing has not been conducted.

In addition, the study included nine background characteristics questions (such age, gender, language, education, leader level, work experience, nursing settings, and nursing location) and seven items mapping EBN support structures (Table 1). The existence of EBN support structures was assessed with the response options Yes, No, and I don’t know.

### 3.4. Data Collection

The data were collected from September through October 2021 in Finland by the NRF using an electronic questionnaire available in both Finnish and Swedish, which are the official languages in Finland. An invitation to participate in the study was sent to the target population by email by the contact persons of the nursing associations and unions. During the data collection process, one reminder was sent to respond to the questionnaire.

### 3.5. Data Analysis

The data were analyzed using IBM SPSS Statistics V.29. The descriptive statistics (frequencies, mean/median, range, standard deviation) were calculated for variables. The variables of organization culture in EBN were re‐coded into three‐point variables (disagree—neither disagree nor agree—agree). Two sum variables (competence and attitudes) were constructed partly based on items from the previously developed Evidence‐Based Practice Process Assessment Scale [22, 23], with permission from the copyright holders. The value of the sum variables was based on the average of the sum variables’ items. The sum variables were examined using frequency distributions, bar diagrams, and location and dispersion indicators. At the analysis stage, several settings with small numbers of participants were combined into an “Other settings” category to simplify descriptive reporting. Respondents could select multiple nursing settings; these data were used solely to describe participants’ background characteristics and were excluded from subsequent group comparisons and inferential analyses. Statistical differences in mean values between groups (background variables or supporting structures and sum variables) were tested by a *t*‐test or a one‐way analysis of variance (ANOVA) with a normal distribution. The normality of the distribution of the variables was assessed using histogram, residuals, and skewness values.

Linear regression models were constructed based on the findings from descriptive and univariate analyses to examine their independent associations of these variables with EBP competence and attitudes. Variables were selected for the regression models according to their statistical significance in the preliminary analyses, in order to minimize the influence of multicollinearity and to maintain parsimony in the exploratory models. The correlations among the variables included in the linear regression analyses were found to be moderate. The variance inflation factor (VIF) values indicated acceptable levels of multicollinearity for both EBN competence (VIF = 1.130–1.823) and EBN attitudes (VIF = 1.068–1.411). No correction for multiple comparisons was applied as analyses were exploratory. The statistical significance limit value for all tests was *p*‐value 0.05. No missing data occurred because the electronic questionnaire used forced responses.

An AI‐based tool (ChatGPT, Copilot) was used to assist with English language editing and proofreading. The tool was used solely for language improvement, and no AI‐generated content was used in the scientific analysis or interpretation.

### 3.6. Ethical Considerations

The study followed guidelines drawn up by the Finnish National Board on Research Integrity (TENK) with good scientific practice and the European Union’s General Data Protection Regulation [24]. The data were authorized to be used by NRF, who obtained the research permits from the trade unions involved in the study. The Research Ethics Committee statement was not required since the study did not touch the physical and mental integrity of the participants [25].

The data were collected using an electronic questionnaire without the identification of participants. The participants were informed in the cover letter about the purpose, voluntary participation, and implementation of the study. A written informed consent to participate in the study was asked. The cover letter was accompanied by the contact details of the researchers carrying out the study for any further questions. The processing of personal data related to the survey was included in the Privacy Policy on the NRF’s website, where participants were directed. The data were treated confidentially throughout the study.

## 4. Results

### 4.1. Participants’ Characteristics

A total of 215 nurse leaders participated in the study; this represents only a small share of the estimated population of Finnish nurse leaders. Therefore, the results may not be fully representative, and all findings should be interpreted with caution. Most of them were female (97.2%), and the mean age was 51.25 years. The majority were front‐line leaders (76.3%), and the rest of them were middle or senior leaders (23.78%). About half of participants had a master’s degree in university or university of applied sciences. The participants’ working experience varied from less than 1 year to 40 years with an average of 11.66 years. Three out of four nurse managers (78.1%) worked in public health organizations. Most of the leaders worked in 24‐h care or hospital settings (Table 2).

**TABLE 2:** Background characteristics of nurse leaders (*n* = 215).

Characteristics	*n*	%	Mean (SD)	95% CI
Age	215	100	51.25 (9.42)	49.98–52.51
Gender				
Female	209	97.2		
Male	6	2.8		
Language				
Finnish	200	93.0		
Swedish	15	7.0		
Education				
Vocational diploma or post‐secondary	41	19.1		
Bachelor’s degree in university of applied sciences or in university	51	23.7		
Master’s degree in university of applied sciences	56	26.0		
Master’s degree in university	56	26.0		
Doctoral or licentiate degree	11	5.1		
Leader level				
Front‐line leader	164	76.3		
Middle or senior leader	51	23.7		
Work experience in leadership (years)	215	100	11.66 (9.25)	10.42–12.90
Organizer of services				
Public	168	78.1		
Private	47	21.9		
Nursing settings^∗^				
University hospital^1^	45	19.9		
Other hospital^1^	48	21.2		
Healthcare center or other equivalent^2^	31	13.7		
Community nursing or hospital at home	18	8.0		
24‐h care	61	27.0		
Other^3^	23	10.2		
Collaborative areas for healthcare and social welfare				
Northern Finland	38	17.7		
Eastern Finland	25	11.6		
Inland Finland	34	15.8		
Western Finland	38	17.7		
Southern Finland	75	34.9		
Åland	5	2.3		

*Note:* CI, confidence interval for mean.

Abbreviation: SD, standard deviation.

^∗^The same respondent has been able to respond to several different nursing settings.

^1^Somatic and psychiatric wards and outpatient clinics.

^2^Wards of the health center, health center, or similar settings.

^3^Undetermined hospital, preventive healthcare, day care or other day‐care unit, emergency care, emergency care, laboratory/sampling, social care, other than defined nursing setting.

### 4.2. EBN Competence and Attitudes

**FIGURE 1:**
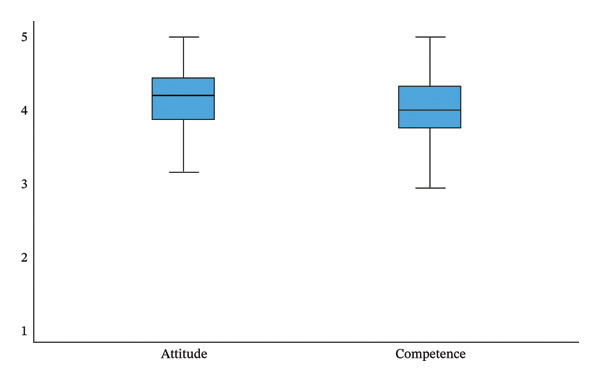
Nurse leaders’ (*n* = 215) attitudes and competence in evidence‐based nursing.

Overall, competence (mean 4.01, SD = 0.476) and attitudes (mean 4.17, SD = 0.413) toward EBN were good among a sample of Finnish nurse leaders, with a slightly higher value for attitudes (Figure 1). There was more variation in the mean of competence (min 2.25 to max 5.00, scale 1–5) than in the mean of attitudes (min 2.94 to max 5.00, scale 1–5) (Table 3). The mean score for competence, considering the different background characteristics, factors related to organizational culture and support structures for EBN, was at least 3.74. This indicates that they rated their competence positively.

**TABLE 3:** Associations between background characteristics of evidence‐based nursing with competence and attitudes among nurse leaders (*n* = 215).

Background characteristics	Competence mean (SD)	*n* ^2^ (95% CI)	F	*p*	Attitudes mean (SD)	*n* ^2^ (95% CI)	F	*p*
Age (years)		0.019 (0.000–0.034)	1.391	0.246		0.020 (0.000–0.078)	1.447	0.230
27–40 (n = 32)	3.91 (0.410)				4.07 (0.430)			
41–51 (n = 69)	4.03 (0.521)				4.13 (0.430)			
51–60 (n = 80)	3.98 (0.447)				4.23 (0.412)			
61–74 (n = 34)	4.13 (0.494)				4.22 (0.354)			
Gender		−0.159 (−0.548–0.229)^∗^	—	0.420		−0.208 (−0.856–0.440)^∗^	—	**0.022**
Female	4.01 (0.480)				4.18 (0.409)			
Male	4.17 (0.274)				3.79 (0.432)			
Language		−0.089 (−0.444–0.266)^∗^	—	0.601		0.189 (−0.151–0.530)^∗^	—	0.254
Finnish	4.01 (0.464)				4.19 (0.394)			
Swedish	4.09 (0.633)				4.00 (0.609)			
Education		0.090 (0.019–0.155)	5.190	**< 0.001**		0.077 (0.012–0.138)	156.02^a^	**0.002**
Vocational diploma or post‐secondary	3.87 (0.513)				4.10 (0.435)			
Bachelor’s degree in university of applied sciences or in university	4.00 (0.464)				4.08 (0.470)			
Master’s degree in university of applied sciences	3.93 (0.409)				4.12 (0.352)			
Master’s degree in university	4.13 (0.465)				4.32 (0.365)			
Doctoral or licentiate degree	4.47 (0.420)				4.42 (0.313)			
Leader level^∗^		−0.259 (−0.406—0.112)^∗^	—	**< 0.001**		−0.163 (−0.292–−0.034)^∗^	—	**0.013**
Front‐line leader	3.95 (0.457)				4.13 (0.414)			
Middle or Senior leader	4.21 (0.485)				4.30 (0.389)			
Work experience in leadership (years)		0.049 (0.000–0.095)	2.150	0.061		0.022 (0.000–0.051)	0.946	0.452
0–5 (n = 54)	3.98 (0.481)				4.16 (0.414)			
5–9.99 (n = 47)	3.90 (0.515)				4.12 (0.419)			
10–14.99 (n = 43)	3.99 (0.456)				4.15 (0.426)			
15–19.99 (n = 27)	4.13 (0.437)				4.25 (0.368)			
20–24.99 (n = 16)	3.94 (0.288)				4.08 (0.537)			
25– (n = 28)	4.22 (0.499)				4.28 (0.339)			
Organizer of services		0.158 (0.004–0.312)^∗^	—	**0.044**		0.166 (0.033–0.297)^∗^	—	**0.015**
Public	4.05 (0.444)				4.21 (0.405)			
Private	3.89 (0.563)				4.04 (0.422)			
Collaborative areas for healthcare and social welfare		0.022 (0.000–0.050)	0.922	0.467		0.011 (0.000–0.027)	0.476	0.794
Northern Finland	3.94 (0.536)				4.23 (0.436)			
Eastern Finland	4.03 (0.363)				4.13 (0.346)			
Inland Finland	4.08 (0.447)				4.21 (0.383)			
Western Finland	4.12 (0.510)				4.11 (0.451)			
Southern Finland	3.96 (0.453)				4.18 (0.426)			
Åland	3.93 (0.753)				4.09 (0.321)			

*Note:* *n* ^2^, Eta‐squared; CI, confidence interval for mean; *F*, ANOVA, *p*, *p*‐value. The bold values indicate statistically significant results.

Abbreviation: SD, standard deviation.

^∗^Mean difference (95 % CI of the difference).

^a^Brown–Forsythe (df2).

### 4.3. Association Between Background Characteristics and EBN Competence or Attitudes

Among background characteristics, higher educational level (*p* = 0.039) was independently and statistically significantly associated with EBN competence (Table 4). In comparison between independent groups, leader level (*p* < 0.001) and the service provider (*p* = 0.047) were also significantly associated with competence in EBN; however, these associations did not remain significant after adjustment in multivariable analyses. A doctoral or a licentiate degree (mean 4.47, SD 0.42) and working at the middle or senior leadership level (mean 4.21, SD 0.49) were connected to better competence. The participants who worked as the public social and healthcare had better competence than those who worked in the private social and healthcare (mean 4.05 [SD 0.44] vs 3.89 [0.56]) (see Table 1).

**TABLE 4:** Linear regression analysis models of factors associated with evidence‐based nursing competence and attitudes among nursing leaders (*n* = 215).

Linear regression models	EBN competence (adjusted R square = 0.232)				EBN attitudes (adjusted R square = 0.151)			
	B (Std. error)	*β*	t	*p*	B (Std. error)	*β*	t	*p*
Education (ref. = Vocation diploma or post‐secondary)	0.059 (0.029)	0.148	2.074	**0.039**	0.043 (0.026)	0.124	1670	0.096
Leader level (ref = front‐line leader vs middle or senior leader)	0.075 (0.080)	0.067	0.937	0.350	0.036 (0.073)	0.037	0.492	0.623
Organizer of services (ref = public vs. private)	−0.122 (0.074)	−0.106	−1.658	0.099	−0.170 (0.068)	−0.170	−2.503	**0.013**
Gender (ref = female vs. male)	—	—	—	—	−0.431 (0.162)	−0.172	−2.666	0.008^∗^
Action Model of Expertise (FinAME model™)^b^	−0.101 (0.052)	−0.137	−1.950	0.053	—	—	—	—
Model for developing EBP (OMEBP)^b^	—	—	—	—	−0.063 (0.049)	−0.096	−1.293	0.197
Care Bundle (The bundle of core activities in nursing)^b^	−0.033 (0.056)	−0.043	−0.587	0.558	−0.045 (0.051)	−0.068	−0.875	0.383
Clinical nurse specialist in nursing^b^	—	—	—	—	−0.033, (0.040)	−0.055	−0.808	0.420
Consultation of Clinical Nursing Sciences Specialists (licentiate, doctoral degrees)^b^	−0.068 (0.039)	−0.115	−1.717	0.087	−0.004, (0.037)	−0.007	−0.096	0.923
The Bank for Best Practices^b^	−0.061 (0.044)	−0.096	−1.383	0.168	−0.105 (0.040)	−0.190	−2.591	**0.010**
In my organization, nurse leaders have an equal opportunity to influence the development of EBN alongside other occupational groups^c^	0.038 (0.047)	0.063	0.801	0.424	0.032 (0.038)	0.062	0.851	0.396
In my organization, nursing staff are supported to establish their practice on evidence^c^	0.085 (0.057)	0.123	1.496	0.136	—	—	—	—
Implementation of EBN is a strategic goal of the organization^c^	0.079 (0.046)	0.132	1.718	0.087	0.008 (0.037)	0.014	0.202	0.840
In my organization, nurse leaders have positive attitudes toward the development of nursing based on evidence^c^	0.072, (0.062)	0.080	1.172	0.243	—	—	—	—
EBN is a competence requirement in my organization (e.g. on the subject of development debate)^c^	0.046 (0.040)	0.082	1.143	0.254	—	—	—	—
We cooperate multiprofessionally in the development of my organization^c^	−0.076 (0.054)	−0.097	−1.410	0.160	—	—	—	—

*Note: B* = unstandardized B, std. Error = coefficients standard error, β = standardized coefficients beta. The bold values indicate statistically significant results.

Abbreviations: EBN, evidence‐based nursing; EBP, evidence‐based practice.

^b^variables (ref. = yes, 1 = no, 2 = I don’t know).

^c^variables (ref. = disargree, 1 = neither disagree nor agree, 2 = agree).

^∗^This result is exploratory only due to the small and unrepresentative sample, VIF EBN Competence = 1.130–1.823, VIF EBN Attitudes = 1.068–1.411.

Among background characteristics, gender (*p* = 0.013) was independently and statistically significantly associated with EBN competence. However, this finding is based on a very small number of male respondents (*n* = 6 vs. *n* = 209 females) and is therefore highly unstable. As such, it is exploratory only and no substantive conclusions can be drawn from this result (Table 4). In comparison between independent groups, education level (*p* = 0.002), leadership level (*p* = 0.013), and service provider (*p* = 0.015) showed significant association with EBN attitudes. However, these associations did not remain significant after adjustment in multivariable analyses. There were slightly better attitudes among middle and senior leaders than front‐line leaders (mean 4.30 [SD 0.39] vs 4.13 [SD 0.41]). Those who had a doctorate and the licentiate degree had better attitudes than others (mean 4.42 [SD 0.34] vs 4.08–4.32). In this study, competence reflected the duration of education in a relatively linear manner, and the spread was reasonably high. Those who worked in public social and healthcare had slightly higher attitudes (mean 4.21 [SD 0.41]) than those who worked in private social and healthcare (mean 4.04 [SD 0.42]) (see Table 1).

### 4.4. Association Between Organizational Factors and EBN Competence or Attitudes

In the linear regression model, the different organizational support structures and organizational culture were not independently associated with EBN competence. However, the FinAME model (*p* = 0.053), consultation with clinical nursing sciences specialists (*p* = 0.087), and having EBN as a strategic goal (*p* = 0.087) showed the strongest associations and were closest to statistical significance (Table 4).

In comparison between independent groups, according to participants’ assessment, the EBN support structures used in the organization, the FinAME model (*p* = 0.009), Care Bundle (*p* = 0.001), and The Bank for Best Practices (*p* = 0.004), and the consultation of Clinical Nursing Science Specialists (licentiate, doctoral degrees) (*p* < 0.001) had a significant association with nurse leaders’ competence in EBN. Regarding organizational culture statements, respondents who rated their organization’s nurse leaders as positive about EBN development (*p* = 0.016) and who felt that nurse leaders had equal opportunities to influence the development of EBN with other professional groups (*p* = 0.001) had better EBN competence. In addition, respondents whose organization supported nursing staff in establishing EBPs (*p* < 0.001), whose organizations had a strategic goal of implementing EBN (*p* < 0.001), and whose organizations considered EBN a competency requirement (*p* = 0.003) had better EBN competence. Respondents who disagreed with the item “We cooperate multi‐professionally in the development of my organization” had better competence, but the difference with respondents who agreed was limited (mean 4.10 [SD 0.35] vs. 4.04 [SD 0.48]) and should be interpreted with caution, as such marginal differences may not persist after adjustment in the multivariable models (see Table 5).

**TABLE 5:** Associations between organization factors (supporting structures and organizational culture) of evidence‐based nursing with competence and attitudes among nurse leaders (*n* = 215).

Organization factors	Competence mean (SD)	*n* ^2^ (95% CI)	F	*p*	Attitudes mean (SD)	*n* ^2^ (95% CI)	F	*p*
Supporting structure of EBN								
Action Model of Expertise (FinAME model™)		0.044 (0.003–0.103)	4.841	**0.009**		0.011 (0.000–0.047)	1.134	0.324
Yes	4.23 (0.474)				4.24 (0.452)			
No	4.06 (0.428)				4.20 (0.430)			
I don’t know	3.91 (0.505)				4.13 (0.385)			
Model for developing EBP (OMEBP)		0.021 (0.000–0.066)	2.232	0.110		0.047 (0.004–0.108)	5.227	**0.006**
Yes	4.16 (0.572)				4.43 (0.320)			
No	4.06 (0.435)				4.20 (0.434)			
I don’t know	3.94 (0.492)				4.10 (0.388)			
Care Bundle (The bundle of core activities in nursing)		0.062 (0.011–0.129)	7.035	**0.001**		0.050 (0.005–0.111)	5.521	**0.005**
Yes	4.34 (0.457)				4.36 (0.431)			
No	4.05 (0.435)				4.23 (0.406)			
I don’t know	3.91 (0.490)				4.08 (0.399)			
Clinical nurse specialist in nursing		0.017 (0.000–0.060)	1.842	0.161		0.035 (0.000–0.090)	3.868	**0.022**
Yes	4.04 (0.438)				4.20 (0.403)			
No	4.02 (0.476)				4.21 (0.423)			
I don’t know	3.85 (0.637)				3.96 (0.407)			
Consultation of Clinical Nursing Sciences Specialists (licentiate, doctoral degrees)		0.083 (0.022–0.156)	9.650	**< 0.001**		0.031 (0.000–0.084)	3.428	**0.034**
Yes	4.14 (0.417)				4.22 (0.434)			
No	4.04 (0.429)				4.22 (0.389)			
I don’t know	3.81 (0.536)				4.06 (0.396)			
Auditing		0.005 (0.000–0.033)	0.549	0.578		0.001 (0.000–0.015)	0.129	0.879
Yes	4.04 (0.474)				4.18 (0.403)			
No	3.97 (0.495)				4.15 (0.446)			
I don’t know	3.95 (0.456)				4.15 (0.415)			
The Bank for Best Practices		0.050 (0.005–0.112)	5.570	**0.004**		0.081 (0.021–0.152)	9.288	**< 0.001**
Yes	4.21 (0.339)				4.37 (0.376)			
No	4.05 (0.486)				4.23 (0.438)			
I don’t know	3.92 (0.489)				4.06 (0.376)			
Organizational culture								
In my organization, nurse leaders have an equal opportunity to influence the development of EBN alongside other occupational groups		0.060 (0.010–0.126)	6.823	**0.001**		0.037 (0.001–0.093)	4.109	**0.018**
Disagree	3.90 (0.530)				4.20 (0.354)			
Neither agree nor disagree	3.85 (0.490)				4.02 (0.380)			
Agree	4.11 (0.428)				4.22 (0.433)			
In my organization, nursing staff are supported to establish their practice on evidence		0.100 (0.032–0.175)	11.722	**< 0.001**		0.013 (0.000–0.052)	1.410	0.246
Disagree	3.74 (0.581)				4.06 (0.403)			
Neither agree nor disagree	3.83 (0.446)				4.14 (0.345)			
Agree	4.11 (0.426)				4.20 (0.433)			
Implementation of EBN is a strategic goal of the organization		0.094 (0.029–0.169)	10.989	**< 0.001**		0.033 (0.000–0.086)	3.583	**0.029**
Disagree	3.80 (0.549)				4.19 (0.409)			
Neither agree nor disagree	3.88 (0.419)				4.04 (0.411)			
Agree	4.13 (0.434)				4.22 (0.408)			
In my organization, nursing staff have positive attitudes toward the development of nursing based on evidence		0.014 (0.000–0.054)	1.515	0.222		0.023 (0.000–0.071)	2.541	0.081
Disagree	4.04 (0.494)				4.26 (0.428)			
Neither agree nor disagree	3.92 (0.425)				4.08 (0.405)			
Agree	4.05 (0.493)				4.20 (0.409)			
In my organization, nurse leaders have positive attitudes toward the development of nursing based on evidence		0.038 (0.001–0.095)	4.200	**0.016**		0.013 (0.000–0.051)	1.374	0.255
Disagree	3.79 (0.429)				4.16 (0.344)			
Neither agree nor disagree	3.84 (0.510)				4.07 (0.405)			
Agree	4.06 (0.462)				4.19 (0.417)			
EBN is a competence requirement in my organization (e.g., on the subject of development debate)		0.053 (0.007–0.116)	5.911	**0.003**		0.017 (0.000–0.059)	1.798	0.168
Disagree	3.91 (0.492)				4.16 (0.382)			
Neither agree nor disagree	3.96 (0.466)				4.11 (0.415)			
Agree	4.16 (0.437)				4.24 (0.437)			
We cooperate multiprofessionally in the development of my organization		0.031 (0.000–0.083)	3.364	**0.036**		0.017 (0.000–0.059)	1.814	0.165
Disagree	4.10 (0.354)				4.23 (0.468)			
Neither agree nor disagree	3.79 (0.486)				4.03 (0.376)			
Agree	4.04 (0.479)				4.19 (0.411)			

*Note:* *n* ^2^ = Eta‐squared, CI = confidence interval for mean, *F* = ANOVA, *p* = *p*‐value. The bold values indicate statistically significant results.

Abbreviations: EBN, Evidence‐based nursing; EBP, evidence‐based practice; SD, standard deviation.

Among the organizational support structures, the use of NFR’s The Bank for Best Practices (*p* = 0.010) was independently and statistically significantly associated with EBN attitudes (Table 4). In comparison between independent groups, according to participants’ assessment, the EBN support structures used in the organization, the implementation of an OMEBP (*p* = 0.006), Care Bundle (*p* = 0.005), presence of the Clinical Nurse Specialists (*p* = 0.022), and possibility of consultation of the Clinical Nursing Science Specialists (licentiate, doctoral degrees) (*p* = 0.034) had a statistically significant connection with attitudes to EBN; however, these associations did not remain significant after adjustment in multivariable analyses. Regarding organizational culture, the nurse leaders who had an equal opportunity to influence the development of EBN alongside other occupational groups (*p* = 0.018) and whose organization’s strategic goal was the implementation of EBN (*p* = 0.029) had a significant connection to attitudes to EBN (see Table 5).

The linear model, which included all statistically significant variables related to competence, explained 23.2% of the variance in EBN competence. Similarly, the linear regression model explained 15.1% of the variance in EBN attitudes (Table 4). These levels of explanation are considered relatively good in nursing research (see Reference [26]). Of the variables included in the model, particularly those related to organizational support structures and organizational culture showed strong and moderate intercorrelations. This may explain why the variables that were significant in the independent group comparisons lost their significance in the linear regression analysis (Table S1).

## 5. Discussion

This study described self‐assessed EBN competence and attitudes, and their association with personal and organizational factors among a sample of Finnish nurse leaders. The relationship between organizational factors (support structures and organizational culture) and nurse leaders’ EBN competence or attitudes has not been previously investigated. In this study, nurse leaders’ EBN competence was examined at a general level, and EBN competence was not determined according to the subdimensions of the EBHC model [2].

In this study, nurse leaders rated their EBN competence and attitudes as good; however, these findings should be interpreted with caution due to the limited representativeness of the sample. Given the well‐known tendency for self‐assessed EBN competence to be overestimated [27, 28], these self‐reported ratings likely represent an optimistic upper bound of nurse leaders’ true EBN competence and attitudes. This may partly explain why previous more detailed studies have reported weaker levels of EBN competence among nurse leaders [6, 9]. Another possible explanation for these differing results is that the previous studies examined more details at EBN competence and focused on front‐line leaders [6, 9].

Regarding EBN attitudes, the results in this sample are consistent with previous studies [6, 9]. Attitudes toward EBN may be positive, even if the competence itself is weak [28]. Own attitudes are often judged to be more positive than those of colleagues [27]. However, to implement the EBN in practice, sufficient competence (including positive attitudes) and support from the organization are needed [2, 7, 8, 12, 29, 30]. Higher educational attainment was the only independent predictor of higher EBN competence among a sample of Finnish nurse leaders. Also, previous studies have found that higher levels of education were associated with EBN competence [14, 30]. Although ANOVA indicated higher EBN competence among middle‐ and senior‐level nurse leaders, leadership level was not an independent predictor in the multivariable model, and this association has not been identified in previous research. However, leader position among advanced practice nurses shows positive association with EBN competence [30].

In this study, work experience was not associated with EBN competence. This is consistent with recent studies, which have not identified work experience as a predictor on competence and have instead found that recent graduates in leadership positions may demonstrate stronger EBN competence [29–32]. Self‐assessed competence is also known to be commonly overestimated [27, 28], which should be considered when interpreting these results.

In this study, no independent statistical associations were found between organizational support structures or organizational culture and EBN competence. However, many of these variables showed strong or moderate intercorrelations, which may explain why independent associations with EBN competence were not identified. One‐way ANOVA nevertheless revealed statistically significant associations between the use of EBN support structures in the organization or organizational culture and nurse leaders’ EBN competence.

This study found that the EBN support structures used in the organization, such as the FinAME model, Care Bundle, and The Bank for Best Practices, as well as the opportunity to consult with Clinical Nursing Science Specialists, had a significant connection with the EBN competence of nurse leaders. The idea of the FinAME model is that the responsibilities for the development of EBN are clearly defined between different levels of clinical nurses, nurse leaders, nursing information management specialists, nurse educators, and researchers [18]. The FinAME model was developed specifically to support the promotion of EBN [18] and includes roles similar to those of mentors and facilitators, who are typically involved in promoting EBN [4]. Mentors and facilitators are considered to play an important role in enhancing EBN competence, yet no previous research has specifically examined their impact. However, this study demonstrated that the use of the FinAME model and Clinical Nursing Science Specialists was associated with nurse leaders’ EBN competence and attitudes.

Among a sample of Finnish nurse leaders, EBN organizational culture was identified as being associated with EBN competence. This finding is consistent with the results of a study on EBN organizational culture and nurses’ EBN competence [33]. In this study, based on the one‐way ANOVA results, EBN competence was related to organizational culture factors, in particular, equal opportunities for nurse leaders to influence EBN development with other professional groups, nurse leaders’ positive attitude toward EBN, recording EBN in the organization’s strategy, and considering EBN as a competence requirement. The relationship between these organizational culture factors and EBN competence has not been studied previously. Huo et al. [33] modeled the mechanism of influence of EBN organizational culture that went from EBN competence developed through EBN adoption intention to EBN adoption and EBN implementation.

In addition to demonstrating that organizational culture and organizational support structures promote nurse leaders’ EBN competence, this study suggests that nurse leaders play a significant role in building an organizational culture that supports EBN and in promoting nursing staff’s EBN competence [7, 8, 15]. The nurse leaders’ competence in EBN is linked to the employees’ view of the evidence implementation [13]. Therefore, supporting the nurse leaders’ competence is relevant to the capability of the entire organization’s EBN. Nurse leaders’ own attitudes and competence toward EBN is a prerequisite for their ability to support the use of evidence by their employees in clinical work [34].

Various support structures and models have been developed to support EBN, but they have not necessarily been implemented or used consistently. However, the use of support structures and models is the first step in achieving reliable, safe, and evidence‐based care [35]. The connections found in this study between nurse leaders’ EBN competence and organizational support structures support the benefits of using support structures. On the other hand, the lack of research information on the benefits of support structures has not contributed to their wider use.

Finally, the findings should be interpreted with caution due to the small sample relative to the estimates national population of nurse leaders and the potential for selection and nonresponse bias. This limits the generalizability of the results.

### 5.1. Strengths and Limitations

The strength of the study was the fact that the participants were from different nursing settings. In addition, all nurse leaders working in social‐ and healthcare Finland had equal opportunity to respond to the survey, which helped to identify a more comprehensive picture for nurse leaders’ EBN competence and attitudes.

This study includes some limitations. First, the use of a self‐assessment instrument may introduce “desirability bias”. Self‐reported EBN competence is known to be systematically overestimated [27, 28], which is relevant given the relatively high mean scores observed in this study. Previous studies using more detailed or objective instruments have reported weaker EBN competence among nurse leaders [6, 9], and thus the current findings should be interpreted with caution. This potential bias was minimized by collecting data without directly identifying information. Another limitation is that the modified ActEBN‐managers instrument used has not been validated. Although internal consistency was excellent, construct validity has not been established, and it is therefore uncertain whether the sum scores fully represent the underlying constructs of EBN competence and attitudes. Until further validation is conducted, results based on this instrument should be interpreted with caution. The third limitation was that the target population represented only a small part (approximately 4%) of the whole population. Response to the study was likely limited by the Covid‐19 pandemic, during which the time resources available to nurse leaders were insufficient to complete the survey. It is also a common phenomenon that survey responses are sparse. The reminder email was used to improve response activity.

In addition to the small sample size, the study is subject to potential selection bias and nonresponse bias. It is possible that those who chose to respond were more interested in or engaged with EBN than nonrespondents. Due to the lack of national sociodemographic data on Finnish nurse leaders, it was not possible to determine whether the sample reflected the broader population. Consequently, the representativeness of the findings cannot be ensured, and the results should be interpreted with caution.

Despite these limitations, the sample provides useful indications of EBN competence and attitudes among the nurse leaders who participated. The findings offer preliminary insights into potential patterns and associations and highlight areas where further research with larger and more representative samples is warranted.

### 5.2. Recommendations for Further Research

The EBN attitudes and competence of nurse leaders should be studied in more detail, for example, by profiling EBN competence levels and identifying which factors are associated with different competence levels. Multivariate logistic regression should be used to investigate which factors have an independent association with EBN competence, and future studies should apply this approach with a larger sample size to strengthen the reliability and generalizability of the findings. Further studies should also aim to validate the ActEBN‐managers version of the instrument.

### 5.3. Implications for Policy and Practice

Although the study found that, in this sample, nurse leaders in Finland generally possess moderately good EBN competence, it also identified variation. Special attention should be given to front‐line leaders, those with lower educational levels, and nurse leaders in the private social and healthcare. In organizations, higher levels of EBN competence among a small of Finnish nurse leaders may be supported by higher education requirements, the use of EBN support structures (such as the FinAME model, care bundles, consultation opportunities, and a bank of good practices), and an organizational culture supportive of EBN (such as by encouraging the implementation of EBN, enabling participation in the development and support of EBN, setting EBN competence standards). At the national level, it may be beneficial to consider a standardized educational requirement for nursing management roles, as nurse leaders play a crucial role in facilitating EBN implementation and promoting the dissemination of evidence within social and healthcare organizations.

## 6. Conclusion

The study provided new information on the EBN competence and attitudes of a small sample of Finnish nurse leaders, and the associations with personal and organizations’ factors. Greater emphasis should be placed on nurse leaders’ EBN competence and organizational factors that support it. Education level emerged as one of the key personal factors associated with positive attitudes and higher competence. Emphasizing education as a key part of a comprehensive EBN approach may be relevant for strengthening implementation nationwide. In addition, organizational EBN support structures and organizational culture were associated with EBN competence and attitudes. Increasing awareness and consistent use of models and support structures, and further developing them, may represent plausible levers to support nurse leaders’ EBN attitudes and competence. However, causal inferences cannot be made from cross‐sectional data. Social and healthcare needs require competent nurse leaders.

## Author Contributions

Saija Ylimäki, Jemina Qvick, Anna‐Maria Tuomikoski, Heidi Parisod, Hannele Siltanen, and Maria Kääriäinen made substantial contributions to conception and design, or acquisition of data or analysis and interpretation of data.

Saija Ylimäki, Jemina Qvick, Anna‐Maria Tuomikoski, Heidi Parisod, Hannele Siltanen, Outi Kanste, and Maria Kääriäinen involved in drafting the article or revising it critically for important intellectual content.

## Funding

This research did not receive any specific grant from funding agencies in the public, commercial, or not‐for‐profit sectors. The research was partly carried out as part of the authors’ employment at the University of Oulu.

Open access publishing was facilitated by Oulun yliopisto, as part of the Wiley—FinELib agreement.

## Disclosure

All authors gave the final approval of the version to be published. Each author should have participated sufficiently in the work to take public responsibility for appropriate portions of the content. All authors agreed to be accountable for all aspects of the work in ensuring that questions related to the accuracy or integrity of any part of the work are appropriately investigated and resolved.

## Ethics Statement

In Finland, the ethical review was not required, but organization‐specific research permits were required and granted by the organizations.

## Conflicts of Interest

The authors declare no conflicts of interest.

## Supporting Information

Additional supporting information can be found online in the Supporting Information section.

## Supporting information


**Supporting Information** Table S1 shows Spearman’s correlation coefficients between evidence‐based nursing (EBN) competence and EBN‐related attitudes, indicating the direction and strength of their associations.

## Data Availability

The data that support the findings of this study are available in the supporting information of this article.
