# Supplementary material for: Evidence‐Based Nursing Competence, Attitudes, and Associated Factors Among Nurse Leaders in Finland: A Cross‐Sectional Study
Source: J Nurs Manag. 2026 May 26;2026:4526663. doi: 10.1155/jonm/4526663 (PMC13213194; doi:10.1155/jonm/4526663)
Supplement: Supplementary file 1 — Supporting Information Table S1 shows Spearman’s correlation coefficients between evidence‐based nursing (EBN) competence and EBN‐related attitudes, indicating the direction and strength of their associations. [file JONM-2026-4526663-s001.docx]

Table S1. Spearman’s correlation coefficients between EBN competence and attitude variables.

|  | ***1*** | ***2*** | ***3*** | ***4*** | ***5*** | ***6*** | ***7*** | ***8*** | ***9*** | ***10*** | ***11*** | ***12*** | ***13*** | ***14*** |
| --- | --- | --- | --- | --- | --- | --- | --- | --- | --- | --- | --- | --- | --- | --- |
| *1 Education* | *1.000* | **0.442*** | 0.022 | -0.079 | -0.185 | -0.116 | -0.131 | -0.176 | -0.049 | 0.080 | 0.044 | -0.018 | -0.047 | -0.075 |
| *2 Leader level* | **0.442*** | *1.000* | -0.035 | -0.133 | -0.157 | -0.067 | -0.219 | -0.252 | 0.202 | 0.140 | 0.093 | 0.035 | -0.010 | 0.007 |
| *3 FinAME model™)* | 0.022 | -0.035 | *1.000* | **0.701**** | **0.536*** | 0.047 | 0.246 | 0.367 | -0.115 | -0.014 | 0.032 | 0.081 | 0.043 | 0.072 |
| *4 OMEBP* | -0.079 | -0.133 | **0.701**** | *1.000* | **0.606**** | -0.033 | 0.247 | **0.418*** | -0.057 | -0.011 | 0.037 | 0.053 | 0.095 | 0.062 |
| *5 Care Bundle* | -0.185 | -0.157 | **0.536*** | **0.606**** | *1.000* | 0.050 | 0.287 | **0.475*** | -0.072 | -0.084 | 0.038 | -0.015 | 0.041 | 0.100 |
| ***6*** *Clinical Nurse Specialist in Nursing* | -0.116 | -0.067 | 0.047 | -0.033 | 0.050 | *1.000* | 0.277 | -0.002 | -0.108 | -0.265 | -0.223 | -0.240 | -0.327 | -0.146 |
| ***7*** *Consultation of Clinical Nursing Sciences Specialists* | -0.131 | -0.219 | 0.246 | 0.247 | 0.287 | 0.277 | *1.000* | 0.289 | -0.209 | -0.210 | -0.156 | -0.032 | -0.151 | -0.123 |
| *8 The Bank for Best Practices* | -0.176 | -0.252 | 0.367 | **0.418*** | **0.475*** | -0.002 | 0.289 | *1.000* | -0.016 | -0.004 | 0.066 | 0.029 | 0.096 | 0.049 |
| *9 In my organization, nurse leaders have an equal opportunity to influence the development of EBN alongside other occupational groups* | -0.049 | 0.202 | -0.115 | -0.057 | -0.072 | -0.108 | -0.209 | -0.016 | *1.000* | **0.546*** | 0.382 | 0.346 | 0.275 | **0.421*** |
| *10 In my organization, nursing staff are supported to establish their practice on evidence* | 0.080 | 0.140 | -0.014 | -0.011 | -0.084 | -0.265 | -0.210 | -0.004 | **0.546*** | *1.000* | **0.502*** | 0.365 | **0.462*** | 0.389 |
| *11 Implementation of EBN is a strategic goal of the organization* | 0.044 | 0.093 | 0.032 | 0.037 | 0.038 | -0.223 | -0.156 | 0.066 | 0.382 | **0.502*** | *1.000* | 0.347 | **0.447*** | 0.311 |
| *12 In my organization, nurse leaders have positive attitudes towards the development of nursing based on evidence* | -0.018 | 0.035 | 0.081 | 0.053 | -0.015 | -0.240 | -0.032 | 0.029 | 0.346 | 0.365 | 0.347 | *1.000* | 0.248 | 0.277 |
| *13 EBN is a competence requirement in my organization* | -0.047 | -0.010 | 0.043 | 0.095 | 0.041 | -0.327 | -0.151 | 0.096 | 0.275 | **0.462*** | **0.447*** | 0.248 | *1.000* | 0.302 |
| *14 We cooperate multi-professionally in the development of my organization* | -0.075 | 0.007 | 0.072 | 0.062 | 0.100 | -0.146 | -0.123 | 0.049 | **0.421*** | 0.389 | 0.311 | 0.277 | 0.302 | *1.000* |
| *EBN = evidence-based nursing,* **** = moderate correlation****,* ***** = strong correlation.*** *All correlations between gender and organizational structure were very weak.* | | | | | | | | | | | | | | |
